# Early Progression with Nodal Involvement Is a Predictor of Early Mortality in Mycosis Fungoides: A Retrospective, Registry-Based Cohort Study

**DOI:** 10.3390/curroncol33100583

**Published:** 2026-09-29

**Authors:** Charalampos Filippatos, Despina Fotiou, Alexandros Briasoulis, Ioannis Ntanasis-Stathopoulos, Evangelos Terpos, Maria Gavriatopoulou

**Affiliations:** 1 Department of Clinical Therapeutics, National and Kapodistrian University of Athens, 11528 Athens, Greece; chfilippatos@med.uoa.gr (C.F.);; 2 Clinic & Laboratory of Pathophysiology, Department of Medicine, National and Kapodistrian University of Athens, 11527 Athens, Greece; desfotiou@med.uoa.gr

**Keywords:** mycosis fungoides, cutaneous t-cell lymphoma, MF, CTCL, nodal involvement, lymphadenopathy, NODE12, prognosis, survival

## Abstract

Nodal involvement is a key indicator of advanced disease in mycosis fungoides (MF). While progression within 24 months is prognostic of survival in other indolent lymphomas, such clinical benchmarks in MF remain underexplored. We aimed to evaluate whether early nodal progression after the initial diagnosis serves as an independent, dynamic clinical predictor of early mortality in patients with MF. This retrospective, registry-based study utilized data from TriNetX. Evaluating post-diagnosis timepoints from 12 to 36 months, the 12-month landmark demonstrated the greatest survival divergence and was selected as the optimal threshold for early nodal progression. Patients without nodal involvement within the first 12 months served as controls. To mitigate immortal time bias, a strict 12-month landmark analysis was employed. Among 25,419 screened patients, 2132 (8.4%) developed nodal involvement within the first 12 months (NODE12). At the 12-month landmark, 21,840 patients were evaluable: 1832 with NODE12 and 20,008 controls. At this landmark, the 5-year overall survival rate was 74.8% for NODE12 patients versus 87.8% for controls (*p* < 0.001). In multivariable Cox regression, NODE12 was an independent predictor of early mortality (aHR = 1.97, 95% CI: 1.77–2.20, *p* < 0.001), alongside advanced age (aHR = 3.58, *p* < 0.001) and lymphocytosis (aHR = 1.69, *p* < 0.001). This survival disadvantage was further confirmed in the PSM sensitivity analysis of 1992 matched pairs (HR = 1.96, 95% CI: 1.66–2.31, *p* < 0.001). In conclusion, NODE12 is a dynamic clinical milestone identifying a high-risk MF phenotype. Similar to POD24 in B-cell lymphomas, NODE12 identifies a high-risk patient subgroup that may benefit from closer monitoring and refined risk stratification.

## Simple Summary

Mycosis fungoides is a rare blood cancer with mainly skin manifestations that typically evolves slowly and has a favorable prognosis. However, it can become dangerous if it spreads to the lymph nodes. While it is known that this spread affects survival, the impact of exactly when it happens has previously been unclear. This study reviewed thousands of patient records and discovered that individuals whose cancer spreads to their lymph nodes within the first year of diagnosis face a twofold higher risk of death compared to those without early spread. This rapid progression and subsequent adverse prognosis likely indicate an aggressive form of the disease. Recognizing this one-year milestone provides clinicians with a simple, practical tool to identify high-risk patients early on. Ultimately, these findings could improve real-world patient care by guiding closer monitoring and personalized treatment decisions for those most at risk.

## 1. Introduction

Mycosis fungoides (MF) is a rare extra-nodal T-cell lymphoma with primary cutaneous involvement, typically characterized by an indolent clinical course with favorable prognosis [1]. However, in approximately 20–25% of cases, disease progression occurs, which is associated with significantly poorer outcomes [2,3]. Advanced-stage MF is clinically heterogeneous and ranges from the development of skin tumors to blood involvement and large cell transformation [3]. Among these indicators of progression, lymph node involvement is the most common and serves as a key component of the established TNMB staging system [4,5,6].

Indeed, advanced-stage MF (clinical stages IIB-IVB) carries a significant mortality burden, with median survival rates under 5 years [4,5]. Furthermore, data from the Prospective Cutaneous Lymphoma International Prognostic Index (PROCLIPI) study recently confirmed that nodal effacement (N3 status) is one of the most critical independent prognostic factors for overall survival (OS) in advanced disease, conferring nearly a twofold risk of mortality [7].

Despite the established prognostic utility of TNMB staging, risk assessment in MF remains largely static, relying primarily on findings at diagnosis. Dynamic clinical milestones occurring during follow-up may provide additional prognostic information and permit timely re-stratification of patients as disease biology evolves. While the adverse prognostic impact of nodal involvement is well established, considerably less is known regarding whether the timing of nodal progression carries independent prognostic significance.

In several indolent B-cell lymphomas, including follicular lymphoma (FL) and Waldenström macroglobulinemia (WM), progression of disease within 24 months of first-line therapy (POD24) has emerged as a robust surrogate marker of biologically aggressive disease, identifying patients at substantially increased risk of early mortality independent of conventional prognostic indices [8,9,10]. Rather than reflecting disease burden alone, early progression appears to identify tumors with intrinsically unfavorable biology that bypass the expected indolent disease course. Whether an analogous temporal landmark exists in MF has not been investigated previously.

Given the central role of lymph node involvement in MF staging and progression, we hypothesized that nodal involvement occurring early after the initial diagnosis may identify a distinct subgroup of patients with biologically aggressive disease and inferior survival. In this context, we present findings from a large-scale, real-world, registry-based analysis assessing the impact of early disease progression with nodal involvement on the survival of MF patients.

## 2. Materials and Methods

### 2.1. Data Source and Study Design

We conducted a retrospective multinational cohort study using data collected on 23 July 2026 from the TriNetX Global Health Research Network, which provided access to electronic medical records (diagnoses, procedures, medications, laboratory values, genomic information) from approximately 158 million patients from 117 healthcare organizations.

### 2.2. The Optimal Threshold Defining Early Nodal Dissemination

To determine the optimal temporal threshold for early nodal progression, a preliminary landmark survival analysis was conducted, evaluating multiple post-diagnosis time points (12, 18, 24, 30, and 36 months). Because standard Receiver Operating Characteristic (ROC) curve analysis for time-dependent variables was not feasible within TriNetX Analytics, the optimal timepoint was defined empirically as the landmark demonstrating the highest risk of death.

### 2.3. Study Population

Newly diagnosed MF patients were identified using ICD-10 codes (C84.0). Patients were required to have at least 12 months of follow-up from the date of initial diagnosis, unless they experienced nodal involvement before that time. Nodal involvement was identified using ICD-10 codes C84.01-C84.08, corresponding to MF involving regional lymph nodes.

As the TriNetX platform aggregates structured administrative data without access to unstructured clinical notes, radiology, or pathology reports, the specific diagnostic modality used to assign these codes cannot be definitively ascertained. Additionally, the database does not provide histopathological staging; therefore, it is not possible to distinguish whether these codes represent benign, reactive dermatopathic lymphadenopathy (N1) or true malignant histological effacement (N2/N3). Therefore, the C84.01–C84.08 codes were utilized as surrogate markers of clinically documented nodal involvement, reflecting the diagnostic judgment of the treating physician at the point of care.

NODE12 was defined as the first instance of nodal involvement occurring within 12 months of the initial MF diagnosis (ICD-10 codes C84.01–C84.08). Patients with MF who developed nodal involvement after the 12-month landmark and those who never received such a coding and were alive at the 12-month landmark formed the control group.

### 2.4. Outcomes

The primary endpoint was OS defined as the time from the 12-month landmark timepoint to death from any cause.

### 2.5. Landmark Analysis

To mitigate immortal time bias and ensure equal survival starting points, a strict 12-month landmark analysis was employed. Survival for all included patients was calculated exclusively from the 12-month landmark following the initial diagnosis of MF. Patients who died or were lost to follow-up prior to the 12-month mark were excluded. Eligible individuals were followed from the index date (the 12-month landmark) until death or loss to follow-up, whichever occurred first.

### 2.6. Sensitivity Propensity-Score-Matched Analysis

A sensitivity analysis was performed using propensity score matching (PSM). Matching variables included age, sex, elevated LDH, lymphocytosis, eosinophilia and the hypopigmented/poikilodermatous MF variant, which are established as independent prognostic markers of survival for MF in the TriNetX database [11]. Moreover, cohorts were matched for race and ethnicity. Matching quality was assessed visually via density plots and quantitatively using standardized differences (SMD), with SMD < 0.100 indicating excellent balance.

### 2.7. Baseline Variables

Baseline demographic characteristics included age at MF diagnosis, sex, race, and ethnicity. Disease-related variables included diagnosis of Sézary syndrome, nodal involvement, erythroderma, folliculotropic MF, hypopigmented MF, and poikilodermatous MF, when available. Laboratory parameters were obtained from the most recent measurement within 6 months prior to MF diagnosis. These included lactate dehydrogenase (LDH), absolute lymphocyte count, and absolute eosinophil count. Elevated LDH, lymphocytosis and eosinophilia were defined according to established literature for MF in TriNetX. Variables with unavailable measurements were not imputed and were handled per the standard of TriNetX Analytics.

Due to the database’s constraints, comprehensive data on non-malignant comorbidities that could act as competing risks for all-cause mortality were not systematically extracted or included in the baseline variable assessment.

### 2.8. Statistical Analysis

Continuous variables were summarized using medians and interquartile ranges (IQR), while categorical variables were presented as frequencies and percentages. OS was defined as the time from the 12-month landmark timepoint to death from any cause. Patients were followed from the index date (the 12-month landmark timepoint) until death or loss to follow-up, whichever occurred first.

OS was estimated using the Kaplan–Meier method via de-identified individual patient-level data, among evaluable subjects. To investigate whether NODE12 is a prognostic factor for survival, hazard ratios (HR) and 95% confidence intervals (CI) were calculated using Cox proportional hazards models. The proportional hazards assumption was assessed using the proportionality test available within the TriNetX Analytics platform. The effect estimate for OS was further a priori adjusted for established prognostic factors of survival for MF in the TriNetX database, including age, gender, elevated LDH, eosinophilia, lymphocytosis and the presence of the poikilodermatous MF variant [11,12,13]. Moreover, exploratory multivariable models were evaluated to account for potential baseline imbalances not captured in the aforementioned adjustment. To account for temporal changes in clinical practice and therapeutic advances, a sensitivity survival analysis was conducted stratifying the study cohort by the calendar year of initial diagnosis (prior to 2015 vs. 2015 and later).

All statistical tests were two-sided, with *p*-values < 0.05 considered statistically significant. Data extraction and analyses were conducted within the TriNetX Analytics platform, with visualizations generated using RStudio/2025.09.2+418 (Posit Software, PBC, Boston, MA, USA). Missing data was handled per default by the TriNetX Analytics platform.

## 3. Results

### 3.1. Verifying the Optimal Early Nodal Progression Timepoint

A total of 25,419 patients were retrospectively screened to identify patterns of early nodal progression at 12-, 18-, 24-, 30-, and 36-month time points after the initial diagnosis of MF. The proportion of patients with early nodal progression at each timepoint was 8.4% (n = 2132), 9.5% (n = 2409), 10.2% (n = 2602), 10.8% (n = 2754) and 11.3% (n = 2875), respectively. Moreover, the total number of patients with nodal progression at any timepoint after the initial diagnosis of MF was 3756 (14.8%). Thus, more than half (56.8%) of these cases occurred within the 1st year after the initial diagnosis and 76.5% of them within 3 years.

In order to evaluate which is the optimal timepoint that defines early nodal dissemination with an impact on prognosis, we conducted a preliminary survival analysis, as an ROC analysis was not feasible within TriNetX. Patients with nodal progression within the 1st year after the initial diagnosis demonstrated the highest risk of death (HR = 2.00, 95%CI: 1.80–2.32), as indicated by the highest Log-Rank X^2^ of 162.037. This acute hazard gradually decreased at subsequent time points, reaching an adjusted value of 1.74 at the 3-year time point, while maintaining statistical significance (Table 1).

### 3.2. Primary Analysis: NODE12 vs. Controls

As progression with nodal involvement within 12 months appeared to confer the worst outcomes among MF patients, this time point was chosen as the threshold for early nodal progression in this manuscript (NODE12).

**Figure 1:**
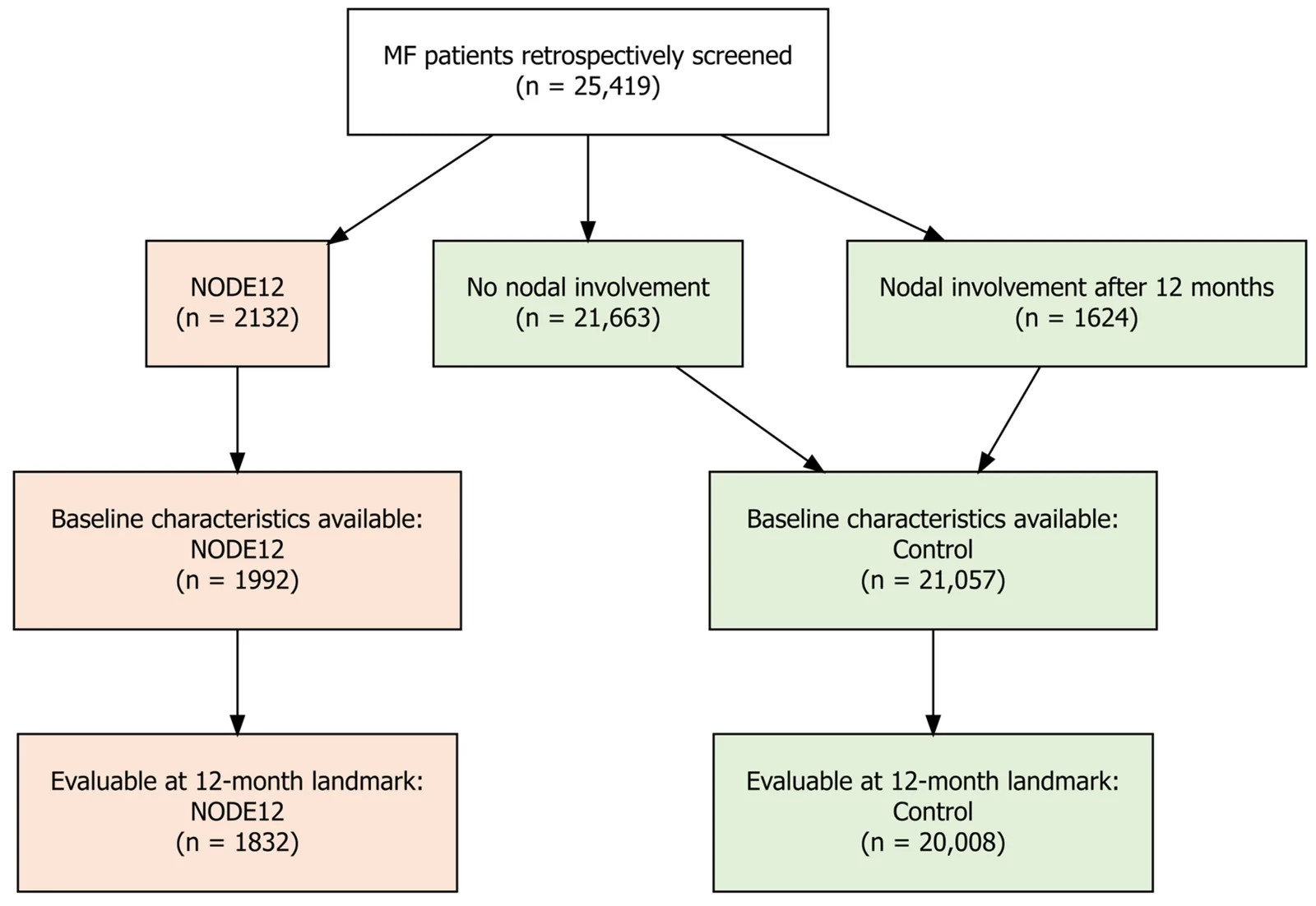
Patient selection flowchart.

A total of 25,419 patients were retrospectively screened, of whom 2132 (8.4%) had NODE12 and 23,287 (91.6%) had no nodal involvement coding through the 12-month landmark. Notably, 1624 (6.4%) patients experienced nodal involvement after the 12-month landmark time point and were included among the controls. Baseline characteristics were accessible for 23,049 patients; 1992 (8.6%) NODE12 and 21,057 (91.4%) controls (Figure 1).

Patients with NODE12 and controls were broadly comparable in age and baseline disease characteristics. The median age at diagnosis was equal between the NODE12 and control cohorts (61 years), and both groups demonstrated the expected male predominance (59.3% vs. 55.5%). Differences were observed in racial and ethnic composition, with a greater proportion of White (64.5% vs. 58.0%), Black or African American (18.7% vs. 14.7%), and Hispanic or Latino (8.2% vs. 5.3%) patients in the NODE12 cohort. Moreover, NODE12 patients exhibited elevated baseline LDH levels (median 208 U/L vs. 144 U/L) and slightly lower absolute lymphocyte counts (1.5 vs. 1.6 × 10^3^\μL) compared to controls. SMDs were <0.10 for most baseline variables, indicating good overall balance, though specific variables such as race, ethnicity, LDH, and ALC presented SMDs slightly above the 0.10 threshold prior to matching (Table 2).

**Figure 2:**
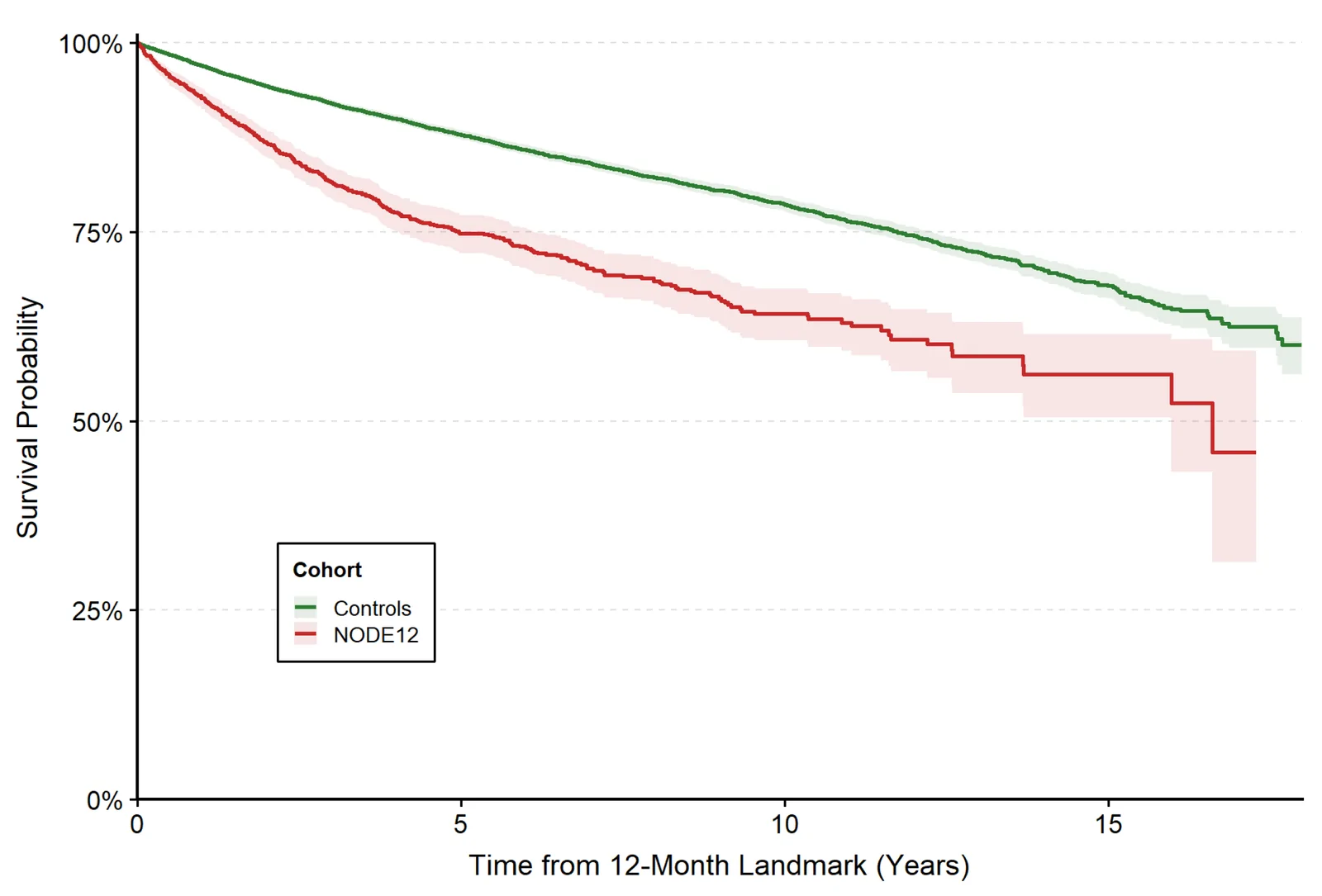
Kaplan–Meier curves for overall survival, total cohort.

The median follow-up from the 12-month landmark for patients in the control group was 3.74 years (IQR 7.36 years), and 3.53 years (IQR 6.08 years) for those with NODE12. During follow-up, a total of 2630 (12.0%) deaths were recorded; 379 (20.7%) among those with NODE12 and 2251 (11.3%) in the control group, with 5-year OS rates of 74.8% (95%CI: 72.2–77.2%) and 87.8% (95%CI: 87.2–88.5%) from the 12-month landmark, respectively (*p* < 0.001, Figure 2).

In the univariate Cox regression, NODE12 was associated with a twofold increase in the risk of death (HR = 2.00, 95% CI: 1.80–2.32, *p* < 0.001) compared with controls. In the multivariate model, NODE12 remained a significant prognostic factor for early mortality (aHR = 1.97, 95%CI: 1.77–2.20, *p* < 0.001). Advanced age (aHR = 3.58, *p* < 0.001) and lymphocytosis also emerged as independent predictors of mortality (aHR = 1.69, *p* < 0.001), followed by male gender (aHR = 1.38, *p* < 0.001) and elevated LDH (aHR = 1.29, *p* = 0.006) (Table 3).

Furthermore, in an exploratory multivariate model incorporating race and ethnicity to account for potential baseline imbalances, NODE12 maintained its independent prognostic significance (aHR = 1.96, 95%CI: 1.75–2.19, *p* < 0.001) (Table S1). The significance of NODE12 was also maintained in sensitivity analyses by the calendar year of initial diagnosis (Tables S2 and S3).

### 3.3. Sensitivity PSM Analysis: NODE12 vs. Matched Controls

**Figure 3:**
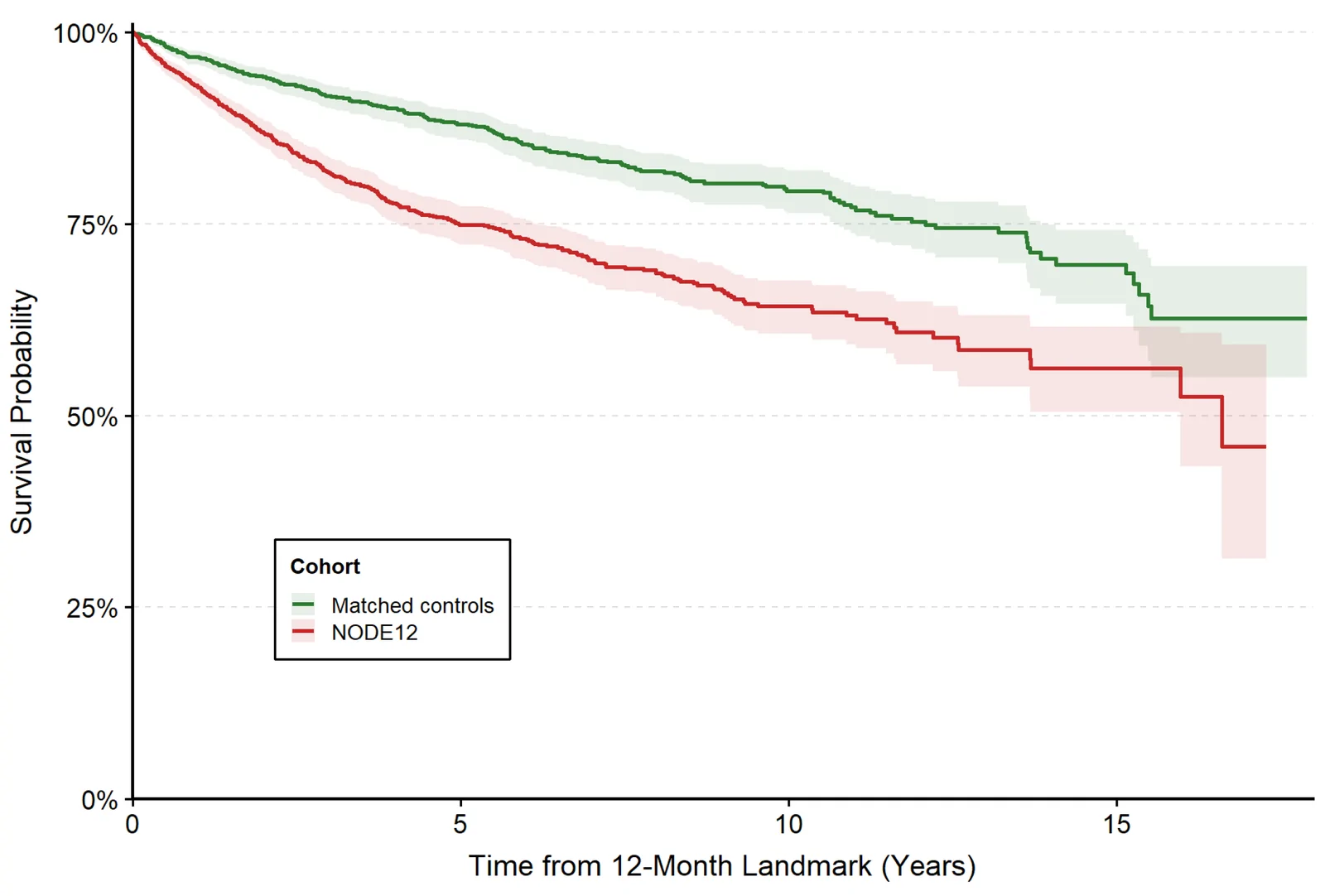
Kaplan–Meier curves for overall survival, matched sub-cohort.

Following PSM, excellent baseline covariate balance was achieved between the NODE12 and matched control cohorts (n = 1992 each), with SMDs < 0.10 for all demographic, disease-related, and laboratory variables (Table S4). Median follow-up from the 12-month landmark was 3.53 years (IQR 6.08 years) in the NODE12 cohort and 4.18 years (IQR 7.32 years) in matched controls. During follow-up, among 1829 evaluable NODE12 patients and 1889 controls, 377 (20.6%) deaths occurred among patients with NODE12 compared with 226 (12.0%) in the matched control cohort. The median OS was 17.63 years in the NODE12 group, while it was not reached in matched controls. The estimated 5-year OS rates from the 12-month landmark were 74.9% (95% CI: 72.3–77.3%) for NODE12 and 88.1% (95% CI: 86.1–89.8%) for matched controls (*p* < 0.001). In this matched sub-cohort, NODE12 remained a statistically significant predictor of early mortality (HR 1.96, 95% CI 1.66–2.31; *p* < 0.001) (Figure 3).

## 4. Discussion

In this large-scale, registry-based analysis of over 20,000 patients with MF, we demonstrate that NODE12 is an independent clinical benchmark for identifying MF patients with poorer outcomes. Our findings demonstrate that the timing of nodal progression is potentially a determinant of early mortality. While the overall indolent nature of MF results in high absolute survival rates, the two-fold increase in mortality risk among NODE12 patients relative to those without nodal involvement at the 12-month timepoint points to an underlying biological aggressiveness.

Notably, the magnitude of the risk associated with NODE12 remained significant even after adjusting (aHR = 1.97, *p* < 0.001) or matching (HR = 1.96, *p* < 0.001) for established prognostic factors and baseline demographic imbalances in this TriNetX population, suggesting that early nodal dissemination represents a distinct clinical phenotype [14]. This marked increase in mortality risk highlights a critical window for intensified surveillance and earlier clinical reassessment, even in cases where the disease was considered borderline early-stage despite findings of lymphadenopathy (e.g., stage IIA) [15,16,17,18].

The prognostic significance of NODE12 likely reflects a distinct evolutionary trajectory of the malignant T-cell clone. In many indolent B-cell malignancies, early progression seems to be synonymous with a ‘high-velocity’ biology that bypasses traditional indolent phases, likely driven by specific genomic alterations or a permissive tumor microenvironment [7,8]. Such early progressors may necessitate tailored therapeutic interventions and a deeper mechanistic understanding of the drivers behind POD24 [19]. By introducing a 12-month cutoff in MF, we provide a temporal window that identifies similar high-risk “early progressors”.

Moreover, our observation of the NODE12 high-risk phenotype could be supported by recent molecular evidence. Miyashiro et al. demonstrated that nearly half of MF patients with histologically ‘uninvolved’ (N1) lymph nodes harbor monoclonal T-cell populations [20]. Notably, they found that this nodal monoclonality serves as an independent prognostic marker (aHR = 3.99), with survival for N1b patients (histologically negative but clonally positive) mirroring that of advanced N2/N3 stages. This suggests that NODE12 may identify the clinical ‘tipping point’ where these early, occult malignant clones manifest as overt disease progression. Consequently, the rapid clinical upstaging observed in our NODE12 cohort may serve as a surrogate for the high-velocity biological escape previously detectable only through molecular clonality assays. Nevertheless, these observations remain hypothesis-generating. Future studies integrating T-cell receptor clonality assays, circulating biomarkers, and molecular profiling of both skin and nodal compartments may help determine whether NODE12 reflects an underlying biologically distinct disease phenotype [21,22,23,24].

These temporal findings also complement the recent development of the Cutaneous Lymphoma International Prognostic Index (CLIPI) by the PROCLIPI consortium. The CLIPI model demonstrated that advanced-stage MF carries a poor 5-year survival rate and identified structural nodal effacement (N3 status) as a critical driver of mortality (HR 1.98) [7]. While CLIPI provides an excellent static assessment of risk at the time of diagnosis, NODE12 provides a dynamic, longitudinal assessment. One possible explanation is that the NODE12 milestone captures the rapid, aggressive clinical transition from reactive dermatopathic lymphadenopathy (N1) to frank malignant effacement (N3). Therefore, incorporating dynamic temporal markers such as NODE12 alongside baseline static scores such as CLIPI could yield a more comprehensive, continuously updated risk stratification model for MF patients.

Finally, our findings expand upon recent large-scale epidemiological data. We previously demonstrated in the total real-world cohort of the TriNetX database, comprising over 25,000 MF patients, that baseline nodal involvement confers a 60% increased risk of mortality (aHR = 1.60) compared to skin-limited disease [11]. In the current study, through a strict 12-month landmark analysis, we demonstrate that early nodal progression (NODE12) further amplifies this mortality risk. This escalation in effect estimates strongly suggests that the timing of nodal dissemination is a more profound determinant of early mortality than its presence at diagnosis.

To our knowledge, this is the first study to establish early nodal involvement as a surrogate marker of mortality in MF in a large, real-world cohort. The application of a strict landmark methodology ensures the stability of our findings and mitigates immortal time bias, while the sensitivity and exploratory analyses further support the robustness of the observed effect estimates.

However, several limitations must be acknowledged. First, while standard clinical practice necessitates histopathological confirmation for a formal diagnosis of MF, the reliance on administrative ICD-10 codes precludes the independent verification of individual pathology reports within the database. Second, the use of the TriNetX registry precludes the availability of TNMB staging [11]. Consequently, the subgroups studied may include patients who experienced other forms of disease progression—such as blood involvement, large cell transformation, or the development of skin tumors—that were not captured by nodal coding, thus confounding our observations. Third, considering the generally indolent disease course of MF, the median follow-up of approximately 4 years across our cohorts may limit the generalizability and reliability of long-term estimates. Fourth, our reliance on administrative ICD-10 codes for lymphadenopathy lacks histopathological validation; therefore, we cannot definitively distinguish benign, reactive dermatopathic lymph nodes (N1) from true malignant infiltration (N2/N3). Fifth, the database’s constraints limited our ability to adjust for specific treatment paradigms and granular patient comorbidities. Because our primary endpoint was all-cause mortality rather than disease-specific survival, we could not definitively account for competing causes of mortality. Therefore, the observed survival trajectories may be partially influenced by age-related or independent comorbid conditions that were not captured in our multivariable models. Furthermore, while the multinational nature of the TriNetX network enhances the generalizability of our findings, it introduces variability in healthcare access and quality of care across different regional nodes. Additionally, temporal variations in systemic therapies were addressed via stratified analyses, yet residual confounding from unmeasured regional treatment disparities remains a limitation inherent to real-world registry studies. Moreover, while our strict 12-month landmark analysis mitigated immortal time bias, it inherently excluded patients who experienced rapid disease progression and died before the 1-year mark. Consequently, our findings reflect conditional survival for patients reaching this temporal milestone and may underestimate the mortality burden of the most hyper-acute progressors. Finally, since the handling of missing baseline variables was governed by the default algorithms of the TriNetX platform and the exact methodologies are not fully disclosed by the network, residual selection bias cannot be excluded if these data were not missing completely at random.

## 5. Conclusions

In conclusion, NODE12 provides a simple, binary clinical milestone that can be easily integrated into standard practice. Similar to POD24 in B-cell lymphomas, NODE12 appears to identify a high-risk patient population that may benefit from closer monitoring and refined risk stratification. Future research should focus on validating NODE12 prospectively and identifying the molecular and genomic drivers that distinguish it from more indolent MF phenotypes.

## Figures and Tables

**Table 1:** Sensitivity analysis of OS outcomes across varying post-diagnosis landmark thresholds.

Landmark	NODE-xx (n)	Control (n)	HR (95%CI)	Log-Rank X^2^
12 months	2132	23,287	2.00 (1.80–2.32)	162.037
18 months	2409	23,010	1.91 (1.70–2.13)	131.010
24 months	2602	22,817	1.84 (1.64–2.07)	107.807
30 months	2754	22,665	1.77 (1.57–2.00)	85.638
36 months	2875	22,544	1.74 (1.53–1.98)	74.316

**Footer:** HR = hazard ratio; CI = confidence interval; NODE-xx = nodal involvement at landmark.

**Table 2:** Baseline population characteristics.

Characteristic	NODE12 (n = 1992)	Control (n = 21,057)	SMD
Age (years)	61 (25)	61 (25)	0.061
Males	1182 (59.3%)	11,686 (55.5%)	0.078
Hispanic or Latino	163 (8.2%)	1120 (5.3%)	0.114
Race			
White	1284 (64.5%)	12,213 (58.0%)	0.133
Black or African American	373 (18.7%)	3092 (14.7%)	0.108
Other/unknown	335 (16.8%)	5752 (27.3%)	-
LDH (U/L)	208 (286)	144 (2620)	0.103
ALC (×10^3^ μL)	1.5 (1.10)	1.6 (1.30)	0.124
AEC (×10^3^ μL)	0.12 (0.18)	0.13 (0.20)	0.074
Hypopigmented or Poikilodermatous MF	62 (3.1%)	554 (2.6%)	0.029
Erythroderma	54 (2.7%)	380 (1.8%)	0.061
Sézary syndrome	38 (1.9%)	204 (1.0%)	0.079

**Footer:** LDH = lactate dehydrogenase; ALC = absolute lymphocyte count; AEC = absolute eosinophil count; MF = Mycosis fungoides; NODE12 = nodal involvement within 12 months of initial MF diagnosis; SMD = standardized mean differences.

**Table 3:** Univariate and Multivariable Cox regression.

Variable in Cox Model	HR	95% CI	*p*-Value	aHR	95% CI	*p*-Value
Univariate			Multivariate			
NODE12 vs. Control	2.00	1.80–2.32	**<0.001**	1.97	1.77–2.20	**<0.001**
Age (≥65 years)	3.11	2.94–3.28	**<0.001**	3.58	3.26–3.94	**<0.001**
Gender (Male)	1.46	1.39–1.55	**<0.001**	1.38	1.26–1.51	**<0.001**
LDH (≥250 IU/L)	1.63	1.49–1.79	**<0.001**	1.29	1.08–1.55	**0.006**
Eosinophilia	1.81	1.59–2.07	**<0.001**	1.14	0.86–1.51	0.375
Lymphocytosis	1.99	1.76–2.25	**<0.001**	1.69	1.33–2.15	**<0.001**
Hypopigmented or Poikilodermatous MF	0.66	0.52–0.84	**0.001**	0.81	0.56–1.18	0.282

**Footer:** HR = hazard ratio; CI = confidence interval; aHR = adjusted HR; NODE12 = nodal involvement within 12 months of initial MF diagnosis; LDH = lactate dehydrogenase; MF = mycosis fungoides; Bold *p*-values denote statistical significance.

## Data Availability

The data analyzed was accessed via the TriNetX Global Health Research Network platform (https://live.trinetx.com/ (accessed on 23 July 2026)) and is available there.

## Supplementary Materials

The following supporting information can be downloaded at https://www.mdpi.com/article/10.3390/curroncol33100583/s1. Table S1: Exploratory multivariable Cox model incorporating race and ethnicity; Table S2: Cox model, initial MF diagnosis ≥2015; Table S3. Cox model, initial MF diagnosis <2015; Table S4: Baseline population characteristics after PSM.
