# Supplementary material for: Early Progression with Nodal Involvement Is a Predictor of Early Mortality in Mycosis Fungoides: A Retrospective, Registry-Based Cohort Study
Source: Curr Oncol. 2026 Sep 29;33(10):583. doi: 10.3390/curroncol33100583 (PMC13703448; doi:10.3390/curroncol33100583)
Supplement: Supplementary file 1 [file curroncol-33-00583-s001.zip › curroncol-4532219-supplementary.pdf]

Table S1. Exploratory multivariable Cox model incorporating race and ethnicity

Variable in Cox model	HR	95% CI	p-value
**Multivariate**			
**NODE12 vs Control**	1.96	1.75-2.19	**<0.001**
Age (≥65 years)	3.54	3.22-3.90	**<0.001**
Gender (male)	1.38	1.26-1.52	**<0.001**
LDH (≥250 IU/L)	1.28	1.07-1.54	**0.008**
Eosinophilia	1.15	0.86-1.52	0.347
Lymphocytosis	1.69	1.33-2.14	**<0.001**
Hypopigmented or Poikilodermatous MF	0.83	0.57-1.20	0.315
White	1.16	1.01-1.33	**0.038**
Black or African American	1.23	1.03-1.47	**0.021**
Hispanic or Latino	0.72	0.54-0.97	**0.033**

Note: LDH = lactate dehydrogenase, MF = Mycosis fungoides

Table S2. Cox model, initial MF diagnosis ≥2015

Variable in Cox model	HR	95% CI	p-value
**Crude, unadjusted hazards**			
NODE12 vs Control (diagnosis ≥2015)	2.23	1.93-2.58	**<0.001**
**Multivariate**			
**NODE12 vs Control** (diagnosis ≥2015)	2.30	2.00-2.65	**<0.001**
Age (≥65 years)	3.22	2.92-3.56	**<0.001**
Gender (male)	1.40	1.27-1.54	**<0.001**
LDH (≥250 IU/L)	1.80	1.51-2.15	**<0.001**
Eosinophilia	1.13	0.89-1.43	0.309
Lymphocytosis	1.83	1.48-2.25	**<0.001**
Hypopigmented or Poikilodermatous MF	0.62	0.45-0.86	**0.005**

Note: LDH = lactate dehydrogenase, MF = Mycosis fungoides

Table S3. Cox model, initial MF diagnosis <2015

Variable in Cox model	HR	95% CI	p-value
**Crude, unadjusted hazards**			
NODE12 vs Control (diagnosis <2015)	1.65	1.39-1.95	**<0.001**
**Multivariate**			
**NODE12 vs Control** (diagnosis <2015)	1.72	1.45-2.03	**<0.001**
Age (≥65 years)	3.08	2.79-3.40	**<0.001**
Gender (male)	1.36	1.23-1.50	**<0.001**
LDH (≥250 IU/L)	1.22	1.03-1.45	**0.022**
Eosinophilia	1.18	0.91-1.52	0.204
Lymphocytosis	1.66	1.34-2.07	**<0.001**
Hypopigmented or Poikilodermatous MF	0.68	0.42-1.09	0.112

Note: LDH = lactate dehydrogenase, MF = Mycosis fungoides

Table S4. Baseline population characteristics after PSM

Characteristic	NODE12 (n=1,992)	Control (n=1,992)	SMD
Age (years)	61 (25)	61 (25)	0.019
Males	1,182 (59.3%)	1,181 (59.3%)	0.001
Hispanic or Latino	163 (8.2%)	168 (8.4%)	0.009
Race			
White	1,284 (64.5%)	1,320 (66.3%)	0.038
Black or African American	373 (18.7%)	354 (17.8%)	0.025
Other/unknown	335 (16.8%)	318 (16.0%)	—
LDH (U/L)	234 (208)	228 (118)	0.089
ALC (x10^3 µL)	1.50 (1.10)	1.62 (1.23)	0.096
AEC (x10^3 µL)	0.12 (0.18)	0.13 (0.17)	0.013
Hypopigmented or Poikilodermatous MF	62 (3.1%)	45 (2.3%)	0.053
Erythroderma	54 (2.7%)	48 (2.4%)	0.019
Sézary syndrome	38 (1.9%)	27 (1.4%)	0.044

Note: LDH = lactate dehydrogenase, ALC = absolute lymphocyte count, AEC = absolute eosinophils count, MF = Mycosis fungoides
